# Serological Array-in-Well Multiplex Assay Reveals a High Rate of Respiratory Virus Infections and Reinfections in Young Children

**DOI:** 10.1128/mSphere.00447-19

**Published:** 2019-09-11

**Authors:** Anna Kazakova, Laura Kakkola, Henna Päkkilä, Tamara Teros-Jaakkola, Tero Soukka, Ville Peltola, Matti Waris, Ilkka Julkunen

**Affiliations:** a Institute of Biomedicine, University of Turku, Turku, Finland; b Department of Biotechnology, University of Turku, Turku, Finland; c Department of Pediatrics and Adolescent Medicine, Turku University Hospital, University of Turku, Turku, Finland; d Clinical Microbiology and Immunology, Turku University Hospital, Turku, Finland; University of Maryland School of Medicine

**Keywords:** adenoviruses, immunoassays, influenza, influenza vaccines, microarray, multiplex, respiratory syncytial virus, upconversion luminescence

## Abstract

Serological assays are used to diagnose and characterize host immune responses against microbial pathogens. Microarray technologies facilitate high-throughput immunoassays of antibody detection against multiple pathogens simultaneously. To improve survey of influenza A virus (IAV), influenza B virus (IBV), respiratory syncytial virus (RSV), and adenovirus (AdV) antibody levels, we developed a microarray consisting of IAV H1N1, IAV H1N1pdm09 (vaccine), IAV H3N2, IBV Victoria, IBV Yamagata, RSV, AdV type 5 hexon protein, and control antigens printed on the bottom of a microtiter plate well. Bound IgG antibodies were detected with anti-human IgG-coated photon-upconverting nanoparticles and measured with a photoluminescence imager. The performance of the microarray immunoassay (MAIA) was evaluated with serum samples (*n* = 576) collected from children (*n* = 288) at 1 and 2 years of age and tested by standard enzyme immunoassays (EIAs) for antibodies to IAV vaccine and RSV. EIAs and MAIA showed substantial to almost perfect agreement (Cohen’s κ, 0.62 to 0.83). Applying MAIA, we found seroprevalences of 55% for IAV H1N1, 54% for IAV vaccine, 30% for IAV H3N2, 24% for IBV Victoria, 25% for IBV Yamagata, 38% for RSV, and 26% for AdV in 1-year-old children (*n* = 768). By the age of 2 years, IgG seropositivity rates (*n* = 714) increased to 74% for IAV H1N1, 71% for IAV vaccine, 49% for IAV H3N2, 47% for IBV Yamagata, 49% for IBV Victoria, 68% for RSV, and 58% for AdV. By analyzing increases in antibody levels not biased by vaccinations, we found a reinfection rate of 40% for RSV and 31% for AdV in children between 1 and 2 years of age.

**IMPORTANCE** The multiplex immunoassay was successfully used to simultaneously detect antibodies against seven different viruses. The developed serological microarray is a new promising tool for diagnostic, epidemiological, and seroprevalence analyses of virus infections.

## Abstract (precis)

The multiplex immunoassay was successfully used to simultaneously detect antibodies against seven different viruses. The developed serological microarray is a new promising tool for diagnostic, epidemiological, and seroprevalence analyses of virus infections.

## INTRODUCTION

Microarray is an emerging technology that provides a versatile platform for many diagnostic and research applications. Protein microarrays are miniaturized versions of traditional assays enabling high-throughput parallel detection of multiple biomarkers in serum samples or other specimens of interest in a single assay (1). To survey seroprevalence of virus-specific antibodies, simultaneous detection of antibodies against multiple viruses is also advantageous, and high-throughput serodiagnostic microarray platforms are under active development for many infectious diseases (2–6).

Respiratory syncytial virus (RSV) and influenza viruses (IAVs) are among the most common causes of respiratory infections in infants and young children, and these viruses continue to infect humans throughout their entire lives, with exceptionally high rates of reinfection. Efficacy of influenza vaccines is continuously monitored for composition adjustments, and many candidate RSV vaccines are under investigation. Human adenoviruses (AdVs) cause respiratory as well as gastrointestinal tract infections, with the highest prevalence in children under 5 years of age (7). The global spread of the most recent pandemic IAV, the current threat of avian influenza, and the continued emergence of novel human viral respiratory pathogens underline the importance of worldwide virus surveillance systems (8). While multiplex PCR assays provide a rapid and specific diagnosis in acute respiratory infections, detection of serum antibodies allows estimating the prevalence of an infection in a population or determination of immune status and antibody responses in vaccine studies. Recently, researchers have started to develop protein microarrays for influenza virus serology (9–14).

For the multiplexed immunoassays, fluorescent dyes are frequently used for detection. NaYF4:Yb^3+^, Er^3+^ photon upconverting nanoparticles (UCNPs) are an exceptional alternative among luminescent labels. UCNPs convert lower-energy near-infrared (980 nm) excitation into higher-energy visible light (550 nm) emission (15). UCNPs are exceptionally photostable, and their anti-Stokes luminescence can be measured free of autofluorescence, which enables very high sensitivity of signal detection. These novel labels have been shown to be suitable for both qualitative and quantitative microarray-based multianalyte assays (16, 17).

In the present study, a multiplex microarray immunoassay (MAIA) was developed for simultaneous detection of serum IgG antibodies against RSV, adenovirus, different influenza virus subtypes or vaccine, and several control antigens. Biotinylated antigens were printed in a 4 × 5 format on the bottom of streptavidin-coated 96-well microtiter plate wells. UCNPs coated with anti-human IgG (anti-hIgG) were used to detect serum IgG antibodies bound to the array spots. The differentiation between and strength of antibody responses against different antigens were achieved based on the position and the intensity of the signal originating from bound UCNPs. The performance of MAIA was validated with a collection of sera previously characterized with in-house enzyme immunoassays (EIAs). We then used MAIA to assess virus-specific immunity in response to natural infection and vaccination in a cohort of young children.

## RESULTS

### Microarray spot signals.

**FIG 1:**
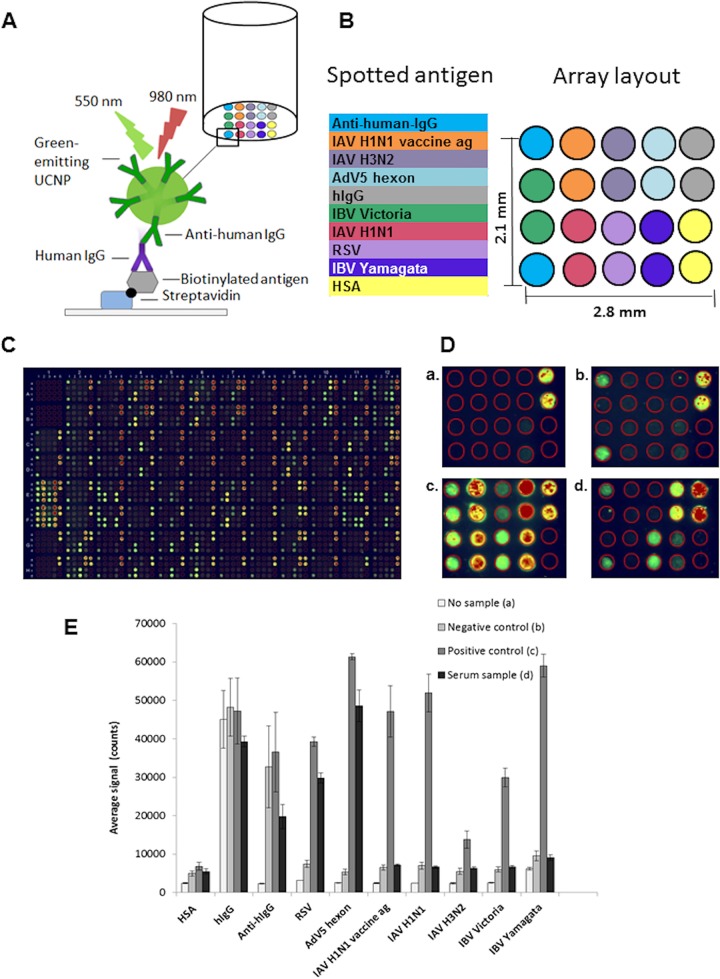
Multiplex array-in-well assay principle, array layout, and fluorescent array-in-well images. (A) Principle of the assay. (B) Array layout. The antigens (ags; IAV H1N1 vaccine, IAV H1N1, IAV H3N2, IBV Yamagata, IBV Victoria, RSV, and AdV5 hexon protein), HSA (human serum albumin; negative control), hIgG (positive control for the detector), and anti-hIgG (positive control for the sample) were printed on the bottom of the streptavidin-coated 96-well microtiter plate. Binding of serum IgG antibodies to the antigens and positive controls was detected by using anti-hIgG coated photon-upconverting nanoparticles (UCNPs). (C) Fluorescent image of an example of the 96-well plate. (D) Fluorescent array-in-well images. (a) An array well without serum sample (reagent background); (b) negative-control serum; (c) positive-control serum; (d) anti-RSV- and anti-AdV IgG-positive sample. Fluorescence colors are related to signal intensity: green corresponds to signal counts of 7,000 to 35,000, yellow to 35,000 to 45,000, and red to 45,000 to 65,000. (E) Average signal counts from no-sample well, negative control, positive control, and a serum sample positive for IgG antibodies against RSV and AdV. Average signal counts were calculated as a mean signal from 2 duplicate spots and 2 duplicate wells (from 4 spots altogether). The error bars indicate the SD of the upconversion luminescence signals.

The principle of the array-in-well assay is shown in Fig. 1A. The differentiation between antigen specificity was achieved based on the signal spot position in the microarray layout (Fig. 1B). The overall visualization of a whole microarray plate is shown in Fig. 1C. A positive signal from hIgG spots indicated binding of UCNP-anti-hIgG without a sample (Fig. 1D, panel a), and positive anti-hIgG spots confirmed the presence of a serum sample (Fig. 1D, panel b). Human serum albumin (HSA) spots served as the control for nonspecific binding. With a positive-control serum sample included, all antigen and control spots (except HSA spots) were positive (Fig. 1D, panel c). A test serum sample with anti-RSV and anti-AdV5 hexon IgG antibodies gave signals from corresponding antigen spots (Fig. 1D, panel d).

Average signal counts detected by the anti-Stokes photoluminescence imager were calculated for each analyte from two replicate spots in two replicate wells (Fig. 1E). The specific signal of each analyte spot was calculated by subtracting the assay background HSA signal from the average signal of the analyte spot. The specific signals were transformed to arbitrary units by linear correlation with a negative (0 units) serum specimen and a positive (100 units) serum specimen used as calibrators.

### Comparison of MAIA and reference EIA results.

For the determination of the cutoff for antibody positivity, microarray results for RSV and IAV vaccine were compared with those of EIA. Altogether, 576 samples (288 samples from 1-year-old children and 288 samples from the same children at 2 years of age) were analyzed by both EIA and MAIA. The area under the receiver operating characteristic (ROC) curve analysis values, 96.5 for RSV and 96.2 for IAV vaccine, indicated good performance of the microarray. Cutoff values of 12 units for anti-RSV and 8 units for anti-IAV vaccine antibodies were determined as the optimal ratios of sensitivity and specificity of the MAIA. We calculated that these cutoff values corresponded to the mean value of negative controls plus 6 standard deviations (SD). The cutoff values for IAV H1N1, IAV H3N2, IBV Yamagata, IBV Victoria, and AdV5 hexon were calculated as the means of negative controls plus 6 SD units, and they were 7, 39, 9, 12, and 5 units, respectively.

**FIG 2:**
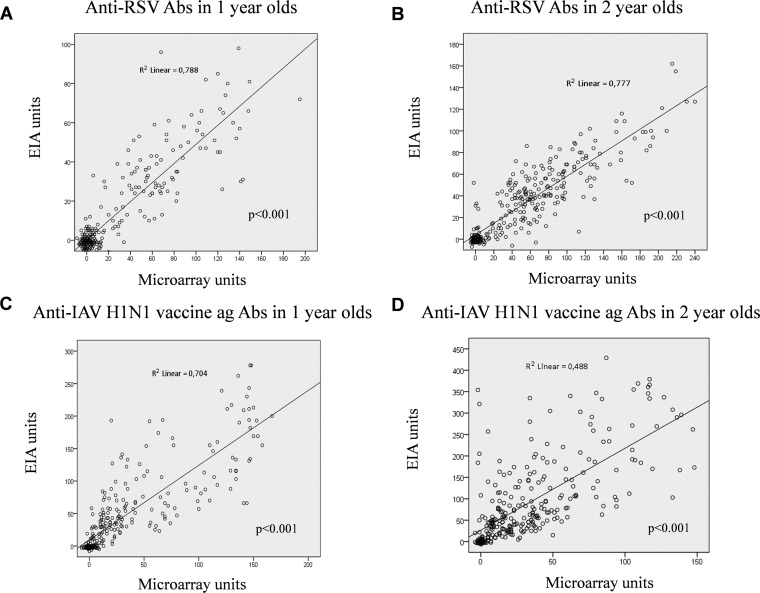
Correlation of the multiplexed MAIA with the reference IgG EIAs (*n* = 288). (A and B) Anti-RSV IgG unit values for serum samples from 1-year-old children (A) and 2-year-old children (B). (C and D) Anti-IAV H1N1 vaccine IgG unit values for serum samples from 1-year-old children (C) and 2-year-old children (D). All 4 correlations were statistically significant (*P < *0.001, two-tailed test).

**FIG 3:**
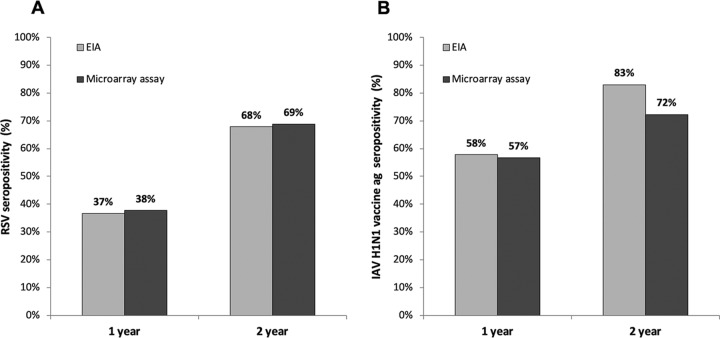
Seropositivity rate comparisons by EIA and MAIA in 1- and 2-year-old children (*n* = 288). (A) RSV; (B) IAV H1N1 vaccine ag.

EIA and MAIA results had a high degree of correlation with each other in both negative and positive serum samples. We observed somewhat higher anti-RSV IgG unit values detected by MAIA than by EIA (Fig. 2A and B), whereas in the anti-IAV H1N1pdm09 vaccine antigen assay, IgG unit values were higher in the EIA (Fig. 2C and D). For a more robust measurement of agreement between the assays, Cohen’s kappa coefficient statistic was used. Comparing the numbers of positive and negative results (Table 1), we found an almost perfect overall agreement between EIA and MAIA for RSV antibodies at 1 and 2 years and for IAV vaccine antibodies at 1 year (Cohen’s κ, 0.829, 0.815, and 0.822, respectively) and substantial agreement for IAV vaccine at 2 years (Cohen’s κ, 0.644). IgG antibodies against RSV were detected in 37% (*n* = 106) and 38% (*n* = 109) of 1-year-old children by EIA and MAIA, respectively (Fig. 3A). By the age of 2 years, 68% (*n* = 196) and 69% (*n* = 199) of children had turned RSV seropositive as detected by EIA and MAIA, respectively. Anti-IAV H1N1pdm09 vaccine antigen IgG were detected in 58% (*n* = 167) of 1-year-old children by EIA and in 57% (*n* = 164) of 1-year-old children by MAIA (Fig. 3B). By the age of 2 years, these seropositivity rates increased to 83% (*n* = 240) and 72% (*n* = 209), respectively.

**TABLE 1:** Comparison of MAIA with the reference EIA IgG assays in determination of the serological status of children (*n* = 288)

IgG assay/age	EIA result	MAIA result (no.)		Cohen’s statistics	
		Positive	Negative	κ	*P* value
RSV/1 yr	Positive	96	10	0.829	<0.001
	Negative	13	169		
RSV/2 yrs	Positive	186	10	0.815	<0.001
	Negative	13	79		
IAV H1N1pdm09/1 yr	Positive	153	14	0.822	<0.001
	Negative	11	110		
IAV H1N1pdm09/2 yrs	Positive	206	33	0.644	<0.001
	Negative	3	46		

### IgG antibody levels and seroprevalences.

**FIG 4:**
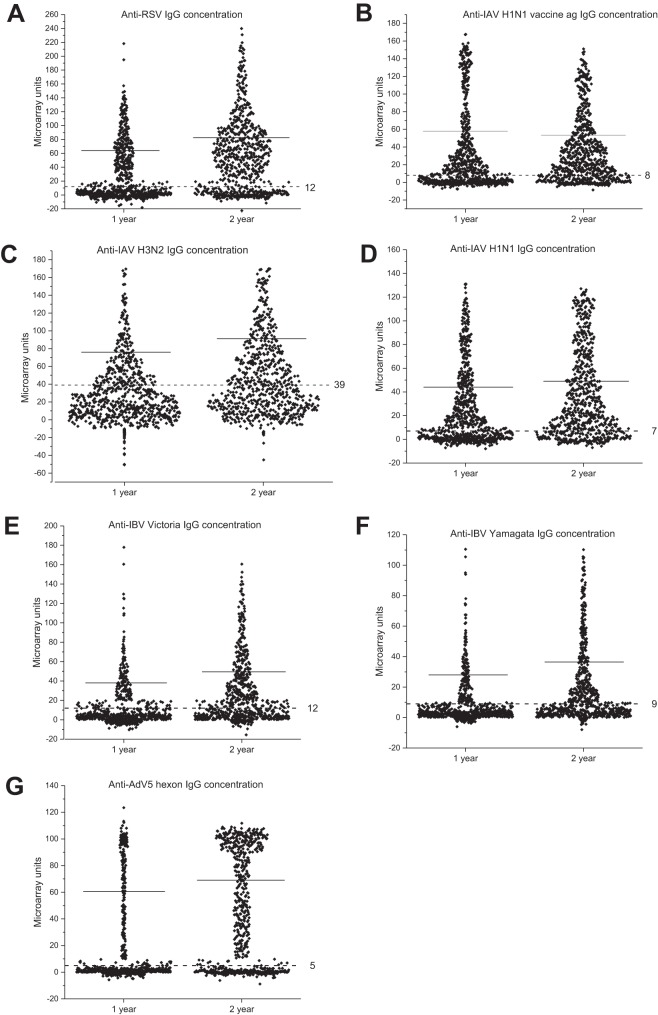
Detection of IgG antibodies reactive with RSV, IAV H1N1 vaccine ag, IAV H1N1, IAV H3N2, IBV Victoria, IBV Yamagata, and AdV5 hexon in 1-year (*n* = 768) and 2-year (*n* = 714) serum samples by the microarray assay. The solid horizontal lines show the mean unit values of seropositive serum specimens; the dotted horizontal lines indicate the cutoff values for each virus antigen. The values are shown as microarray units (MAIA), calculated in relation to the values for negative-control specimens (MAIA unit value set as 0) and a highly positive serum specimen (MAIA unit value set as 100).

**FIG 5:**
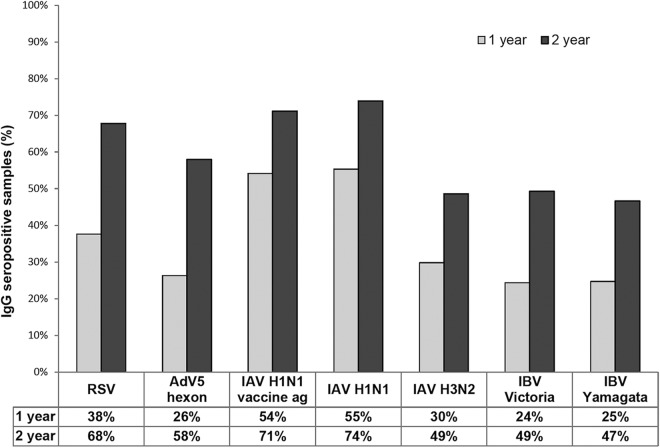
Seropositivity rates for antibodies against all tested antigens (RSV, AdV5 hexon, IAV H1N1 vaccine ag, IAV H1N1, IAV H3N2, IBV Yamagata, and IBV Victoria) in serum specimens collected from 1-year-old (*n* = 768) and 2-year-old (*n* = 714) children by the microarray assay.

Figure 4 shows the distribution of IgG unit values, measured for the seven different virus antigens on the microarray, in all serum samples collected from 1-year-old (*n* = 768) and 2-year-old (*n* = 714) children. Calculated cutoffs clearly discriminate seropositive from seronegative samples. Between the time points, an increase of mean IgG levels was observed against all virus antigens, except the IAV vaccine. The rise in mean IgG levels was likely due to both reinfections and a stronger response to primary infection after the first year of life. The seroprevalence of antibodies increased against all viruses between 1 and 2 years. At 2 years of age, it had increased 1.79-fold for RSV, 2.23-fold for AdV, 1.31-fold for IAV vaccine, 1.35-fold for IAV H1N1, 1.63-fold for IAV H3N2, 2.04-fold for IBV Victoria, and 1.88-fold for IBV Yamagata (Fig. 5).

### High IgG antibody levels develop after reinfection.

**FIG 6:**
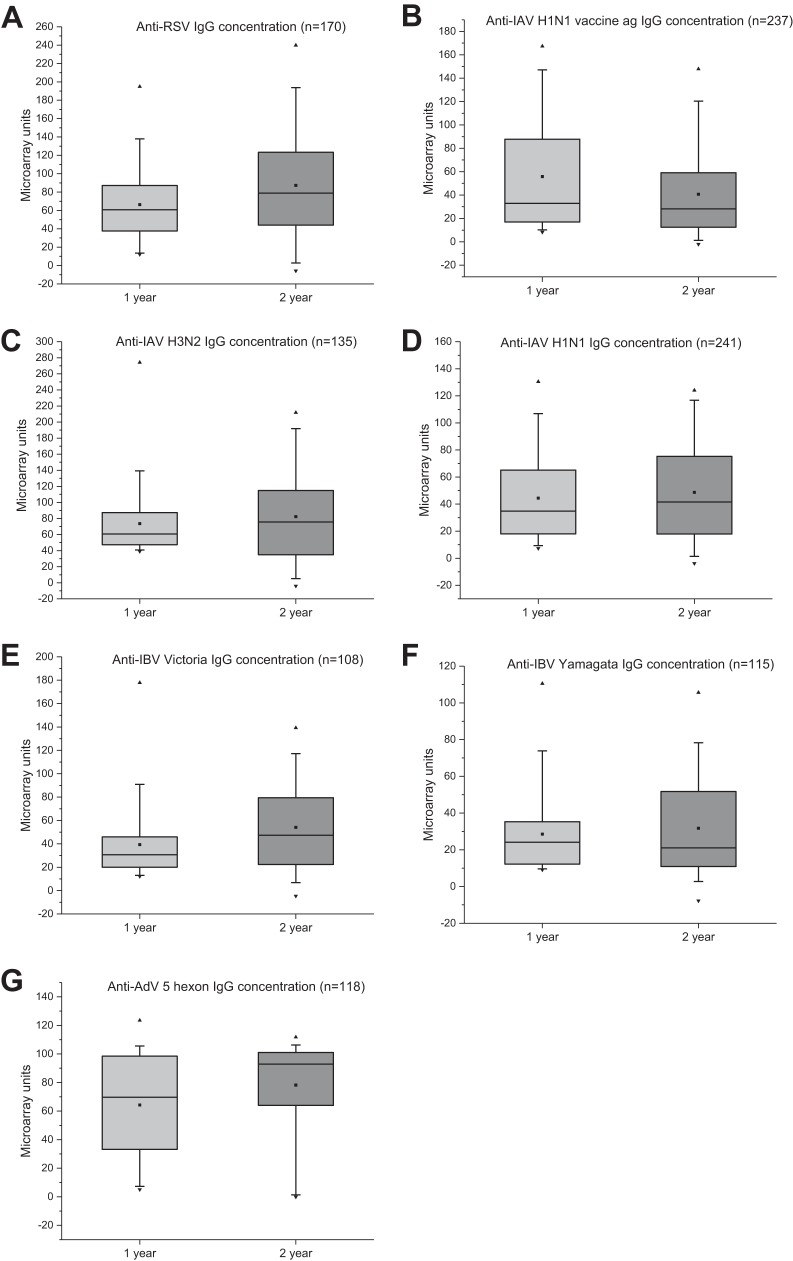
Follow-up of mean IgG antibody levels for RSV, IAV H1N1 vaccine ag, IAV H1N1, IAV H3N2, IBV Victoria, IBV Yamagata, and AdV5 hexon protein in children who were seropositive at 1 year (among 450 individuals for whom both 1-year and 2-year samples were available). The boxes represent the interquartile range (25 to 75%), the whiskers indicate 1st and 99th percentiles, the horizontal lines inside boxes show a median, the squares inside boxes show a mean, and the triangles show minimum and maximum. *n*, number of serum specimens.

**FIG 7:**
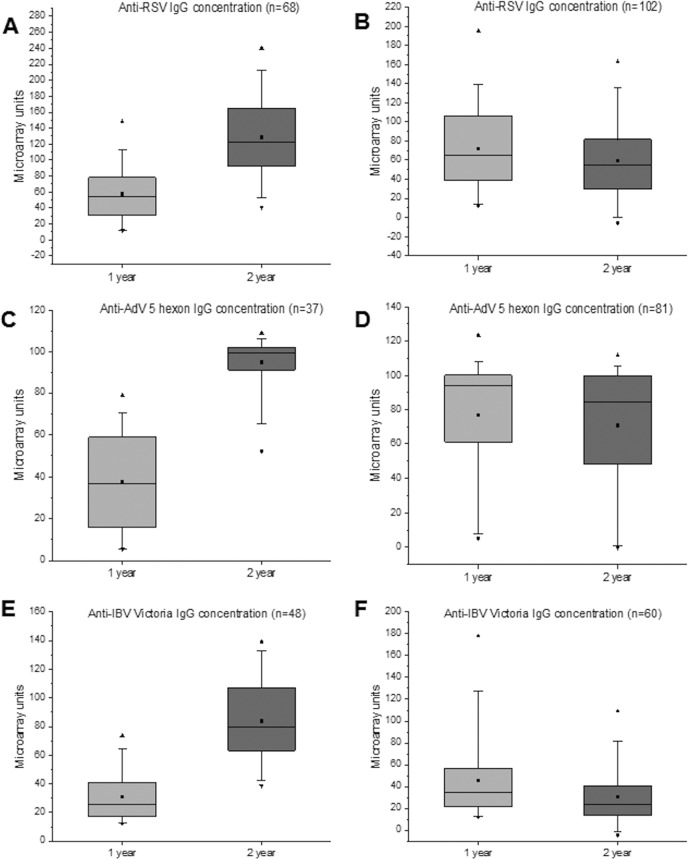
Levels of RSV-, AdV5 hexon-, and IBV Victoria-reactive IgG in children who were IgG seropositive in the corresponding assay at 1 year (*n* = 170, *n* = 118, and *n* = 108, respectively). Children were separated into two groups based on their likely reinfection. (A, C, and D) Mean IgG levels in children with >25 MAIA units increased at the age of 2 years compared to the 1-year sample (likely reinfection between 1 and 2 years). (B, D, and F) Mean IgG levels in children showing increases of ≤25 MAIA units during follow-up. The boxes indicate the interquartile range (25 to 75%), the whiskers 1st and 99th percentiles, the horizontal lines inside the boxes the median, the squares inside boxes the mean, and the triangles the minimum and the maximum.

To further estimate seropositivity rates, antibody levels, and possible reinfection rates, serum samples collected at 1 and 2 years of age from 450 individuals were analyzed (Fig. 6). Children who were seropositive for RSV, AdV, or IBV Victoria by the age of 1 year showed increased mean antibody levels at the age of 2 years, suggesting a high rate of reinfection. They were divided into 2 groups based on the change in the corresponding IgG level. An increase of >25 MAIA units was considered an indication of reinfection. Of the 170 children who had acquired RSV infection by the age of 1 year, 68 (40%) were likely to have had a reinfection by the age of 2 years (Fig. 7A), with an increase of the mean anti-RSV IgG antibody levels from 58 to 129 MAIA units. The antibody levels of the group of 102 children without serological evidence of a reinfection (Fig. 7B) decreased slightly, from 72 to 60 MAIA units (*P = *0.030). Of the 118 children who had acquired AdV infection by the age of 1 year, 37 (31%) likely had a reinfection by the age of 2 years (Fig. 7C), with an increase of mean antibody levels from 37 to 95 MAIA units. The remaining 81 anti-AdV antibody positive individuals showed no change in the mean IgG levels (76 MAIA units versus 70 [*P = *0.262] [Fig. 7D]). Of the 108 children who had acquired IBV infection by the age of 1 year as analyzed by Victoria strain-specific reactivity, 48 (44%) were reinfected by the age of 2 years, with an increase of antibody levels from 31 to 84 MAIA units (Fig. 7E). The other group of 60 children showed some decrease in corresponding antibodies, from 46 to 30 MAIA units (*P = *0.007) (Fig. 7F). The responses to Yamagata antigen were weaker, with an increase from 23 to 52 MAIA units in the former group of 48 children, including only 17 individuals with a qualified increase of >25 MAIA units. Interestingly, the groups of children without evidence of reinfection showed 1.24-, 2.05-, and 1.48-fold-higher mean antibody levels after primary infection than the groups showing serological evidence of reinfection with RSV, AdV, and IBV Victoria, respectively. It is likely that higher antibody levels at least partly protected these children from reinfections.

### RSV IgG seropositivity and reinfection rates by RSV-specific RT-PCR.

From the studied group analyzed by both EIA and MAIA (*n* = 288), 183 children were followed more intensively for respiratory tract infections. Nasal swab specimens were taken during symptoms of infection and analyzed by reverse transcription-PCR (RT-PCR) for the presence of RSV RNA. Among the 183 children, 63 had at least one RT-PCR-confirmed RSV infection. A relationship between PCR-confirmed RSV infection and RSV IgG antibody levels detected by EIA has been published previously (18). Among the RSV RT-PCR-positive individuals by the age of 13 months, 5% (2/40) were IgG seronegative as detected by MAIA. By 2 years of age, only 1 child out of 63 (1.6%) RSV RT-PCR-positive individuals remained RSV seronegative by MAIA (Table 2).

**TABLE 2:** Relationship between detection of RSV RNA in nasal swabs during respiratory infections before 24 months of age and RSV IgG antibody levels in sera at 13 and 24 months of age

Case no.	Mean age (range) in mo at RSV RNA detection(s) (<13 mo)	RSV IgG at 13 mo		Mean age (range) in mo at RSV RNA detection(s) (13–24 mo)	RSV IgG at 24 mo		Change in mean MAIA units
		Mean MAIA units (range)	Seropositivity rate or status		Mean MAIA units (range)	Seropositivity rate or status	
Cases with a single RT-PCR-confirmed RSV infection episode							
1–29	7.7 (0.8–13.5)	63 (11–134)	97%	Not detected	79 (0–240)	97%	+16
30–52	Not detected	5 (0–34)	13%	17.1 (13.1–24.7)	83 (35–215)	100%	+78
Cases with two RT-PCR-confirmed RSV infection episodes							
53	1.9	82	Positive	25.4	187	Positive	+105
54	2.6	16	Positive	18.3	58	Positive	+42
55	2.8	7	Negative	23.9	165	Positive	+158
56	4.2	64	Positive	16.0	84	Positive	+20
57	6.1	74	Positive	17.1	75	Positive	+1
58	9.7	60	Positive	23.1	191	Positive	+131
59	11.2	59	Positive	21.2	108	Positive	+49
60	2.3; 12.3	54	Positive	Not detected	89	Positive	+35
61	2.9; 9.7	24	Positive	Not detected	16	Positive	−8
62	4.1; 10.4	68	Positive	Not detected	131	Positive	+63
Case with three RT-PCR-confirmed RSV infection episodes							
63	5.8; 7.4	82	Positive	18.3	126	Positive	+44

## DISCUSSION

In the present study, a multiplex serological MAIA was developed for simultaneous detection of serum IgG antibodies against IAV H1N1 vaccine antigen (pandemic A/California/7/2009 virus), IAV H1N1, IAV H3N2, IBV Yamagata, IBV Victoria, RSV, and AdV5 hexon protein. To validate the developed assay, serum specimens collected at 1 and 2 years of age from 288 individuals were tested by MAIA and by reference in-house IgG antibody EIAs against RSV and IAV H1N1 vaccine antigen. The detection of RSV- and IAV-specific antibodies by the multiplex MAIA was in very good agreement with results obtained by EIA reference methods. The data indicate that the developed serological microarray proved to be a reliable tool for analyzing virus-specific humoral immunity in response to natural infection and vaccination in young children.

During our study, and as a previously described pattern, a major RSV epidemic peak was observed in Finland every other winter (2010 and 2012), with minor epidemic peaks occurring between the major ones (2009 and 2011) (7). Thus, most of our samples covered at least one RSV epidemic season. Based on anti-RSV IgG antibody seroprevalence analysis, at least 38% of young children were infected with RSV during their first year of life and 68% by the age of 2 years. Interestingly, the seroprevalence data corresponded extremely well with our previous findings from a smaller cohort analyzed by EIA (18). Reinfection led to clearly increased serum anti-RSV IgG antibody levels. It was also of interest that the children, who had relatively high anti-RSV antibody levels at 1 year of age, did not show any further increase in antibody levels and thus were likely protected against a reinfection between 1 and 2 years of age. During the study period, various AdV types were circulating endemically, but AdV-B3 caused an outbreak in the second half of 2010 (19). Hexon protein is known to be highly cross-reactive between AdV serotypes. In our seroprevalence analysis, at least 26% of young children were infected with AdV during their first year of life and 58% by the age of 2 years. About 31% of children who had undergone an AdV infection during their first year of life were reinfected by the age of 2 years. Reinfection led to the development of relatively high anti-AdV antibody levels. Those children who had no serological evidence of reinfection had high anti-hexon antibody levels already at the age of 1 year.

The pandemic IAV H1N1pdm09 was the prevalent IAV in Finland during the collection of 1-year samples (2009 to 2011), cocirculating with IBV in the 2010–2011 epidemic season. The 2011–2012 season was predominated by IAV H3N2. In Finland, the pandemic vaccination coverage was ca. 80% in children aged 6 to 23 months (20). In our study, 54% of children were seropositive against IAV H1N1 vaccine antigen by the age of 1 year and 71% by the age of 2 years. Similar seropositivity rates were detected with partially purified whole H1N1 IAV (55% and 74%, respectively). The H1N1 vaccine antigen and the epidemic IAV strain used in the analysis are genetically very close to one another, which explains the similar seropositivity rates. In contrast to all other antigens, the levels of IgG antibodies against the two IAV antigens were similar or slightly decreased by the age of 2 years, suggesting that anti-H1N1 antibodies (at the age of 1 year) protected the children against IAV infection or reinfection. Increased seropositivity by the second year was likely due to better vaccination coverage by the age of 2 years. Our data suggest that the high seropositivity rate of H1N1 IAV-specific antibodies was due to an effective vaccination campaign, and thus, we could not analyze the rate of natural IAV infection in our cohort (21).

We also included influenza A H3N2 virus into our analyses even though the virus became epidemic only during the winter of 2011–2012 among the studied epidemic seasons. In our study, 30% of children were seropositive against IAV H3N2 by the age of 1 year and 49% by the age of 2 years. This was likely due to cross-reactivity of conserved internal proteins and vaccinations with the seasonal trivalent influenza vaccine recommended for all 6- to 35-month-old children during the study years. The seasonal vaccination coverage was variable; in the study population, it ranged from 22 to 47%. However, IAV H3N2 antigen gave relatively weak reactivity in the array-in-well assay, thus limiting the reliability of the cutoff value discriminating seropositive and seronegative serum specimens.

There are two evolutionary lineages of human IBVs, Victoria and Yamagata. For the study period, the IBV included in the seasonal vaccine was from the Victoria lineage, matching the strains predominating during that time (22). In our study, 24% and 25% children were seropositive against IBV Victoria and Yamagata antigens by the age of 1 year and the seropositivity rose to 49% and 47% by the age of 2 years, respectively. Out of those children who had a primary response to IBV Victoria by the age of 1 year, 44% showed a significant increase in antibody levels, indicating a likely IBV reinfection or reimmunization. Most of our 2-year samples were collected in 2011, suggesting that there were a relatively high number of IBV Victoria strain-specific infections. It has to be pointed out that our assay is not IBV lineage specific, since IBVs of different lineages show very high homology for the viral internal proteins and relatively high homology for the envelope glycoproteins. Our results indicate that lineage-specific antigens enable higher sensitivity in the detection of changes in the antibody levels.

In the present study, we have developed a multiplexed serological microarray assay and demonstrated its excellent performance for the screening of a large number of serum specimens collected from young children for the presence of IgG antibodies against several viruses or viral antigens. The data presented show that young children are highly susceptible to respiratory infections and the frequency of reinfections is relatively high. With the developed microarray method we can obtain quantitative data and estimate not only sample seropositivity but also IgG antibody levels and changes between serial serum specimens and possible reinfection rates. The overall performance and technical features of the assay showed that the platform supports viral proteins, whole viruses, and viral vaccine as antigens. This microarray assay can be used as a diagnostic test and for large-scale epidemiological and seroprevalence studies to simultaneously detect immune responses against multiple antigens. In addition, the method is readily suitable for detecting vaccine-induced immune responses in vaccine efficacy studies.

## MATERIALS AND METHODS

### Serum samples.

Serum samples were obtained from children who participated in an interdisciplinary, prospective observational birth cohort study called Steps to the Healthy Development and Well-being of Children (the STEPS study) (23). The study protocol was approved by the Ethics Committee of the Hospital District of Southwest Finland (reference number 16/180/2008). Parents of participating children gave their written, informed consent. In this study, 1,482 serum samples collected from the children at the ages of 13 months and 24 months were tested for the presence of IgG antibodies against seven different viral antigens. In a previous study (18), a subcohort of serum specimens that included all three serum samples collected at 13, 24, and 36 months of age from a total of 288 children was analyzed for the presence of anti-RSV IgG by an in-house enzyme immunoassay (EIA). All serum samples from the subcohort of 288 children were analyzed also by an in-house EIA for IgG antibodies against IAV vaccine antigen (data not shown). For the EIA, IAV H1N1pdm09 vaccine split-virus antigen of purified, inactivated A/California/07/2009 virus (Pandemrix vaccine; GlaxoSmithKline Biologicals S.A., Germany) was dissolved in phosphate-buffered saline (PBS; pH 7.2) at a concentration of 1.25 μg/ml and adsorbed onto the wells of polystyrene microtiter plates (Combiplate, 96-well format; Thermo Scientific, USA) using 100 μl/well. All EIAs were performed essentially as described previously (24).

### Antigens and controls.

The microarray included seven different viral antigens and three controls. The antigens were partially purified IAV H1N1, IAV H3N2, influenza B virus (IBV) Victoria, IBV Yamagata, and RSV whole viruses, purified AdV5 hexon protein, and IAV H1N1pdm09 vaccine antigen (Pandemrix, GlaxoSmithKline Biologicals S.A., Germany). The controls included purified human IgG (hIgG) (Sigma-Aldrich, St. Louis, MO) as a positive control for the anti-hIgG-coated upconverting nanoparticles (UCNPs), rabbit anti-hIgG (Thermo Scientific, Rockford, IL) as a positive control for the serum samples, and human serum albumin (HSA; Sigma-Aldrich) as a negative control to determine the extent of nonspecific binding in the assay.

The AdV species C type 5 prototype was originally obtained from the Centers for Disease Control and Prevention (Atlanta, GA). It was cultivated in HeLa cells, and hexon protein was purified by anion-exchange chromatography as previously described (25). Influenza viruses A/Turku/10/2009 (H1N1), A/Finland/208/2012 (H3N2), B/Finland/51/2011 Victoria-like lineage, and B/Finland/58/2011 Yamagata-like lineage were propagated in the MDCK cell line and partially purified by pelleting through a sucrose cushion (30%, wt/wt). The pellets were resuspended in a small volume of phosphate-buffered saline (pH 7.3), aliquoted, and stored at −60°C. RSV subgroup A (Randall strain) was propagated in Vero cells. RSV antigen purification was performed essentially as described previously (25).

### Biotinylation of antigens and capture proteins.

Capture antigens were biotinylated prior to immobilization onto streptavidin-coated plates with 15- to 40-fold molar excesses of biotin isothiocyanate (BITC) in 50 mM carbonate buffer (pH 9.8), and the reaction proceeded for 4 h at room temperature in rotation. BITC was synthesized as described previously (26). Biotinylated antigens were purified from free biotin either with Sephadex NAP-10 and PD-10 columns (GE Healthcare Bio-Sciences AB, Uppsala, Sweden) using 50 mM Tris base (pH 7.75), 150 mM NaCl, and 0.05% (wt/vol) NaN_3_ or with an Amicon Ultra 10K centrifugal filter device (Merck Millipore) using PBS (pH 7.4) and 0.05% (wt/vol) NaN_3_.

### Synthesis of UCNP and surface modification.

NaYF4:Yb^3+^, Er^3+^ upconverting nanoparticles were synthesized and functionalized with poly(acrylic acid) (PAA; molecular weight [MW], 2,000; Sigma-Aldrich) using protocols described earlier (19, 27). Rabbit anti-human IgG (Dako, Denmark) was conjugated onto UCNPs according to a previously published protocol (28), with slight modifications. The carboxyl groups on the surface of the PAA-functionalized UCNPs were activated using 5 mM 1-ethyl-3-(dimethylaminopropyl)carbodiimide and 30 mM sulfo-*N*-hydroxysuccinimide. The antibody conjugation was done in 250 μl using 0.083 mg of antibodies for the conjugation of 2.5 mg of UCNPs.

### Array-in-well fabrication.

White flat-bottom 96-well streptavidin-coated KaiSA microplates were obtained from Kaivogen Co. (Turku, Finland). Biotinylated antigens were printed to streptavidin-coated wells into an array format using a noncontact microarrayer Nanoplotter 2.1 (GeSiM, Germany) with 70% humidity, 80-V voltage, 50-μs pulse, 100-Hz frequency, and 2 droplets/spot. Viral antigens (*n* = 7) and controls (*n* = 3) were diluted in printing buffer (PBS [pH 7.4], 10% glycerol [vol/vol]) and printed in duplicates forming a 5 × 4 spot array in the well. The printing concentration of biotinylated whole-virus antigens IAV H1N1, IAV H3N2, IBV Victoria, IBV Yamagata, and RSV as well as biotinylated HSA was 400 μg/ml. Protein concentrations of other biotinylated antigens were 200 μg/ml for IAV H1N1pdm09 vaccine, 150 μg/ml for AdV5 hexon protein, 100 μg/ml for anti-hIgG, and 50 μg/ml for hIgG. The antigen concentrations of the controls were optimized so that their spots would be clearly visible but not outshine the virus antigen spots. Two replicate spots were printed for each analyte.

After the printing, the wells were blocked with 100 μl of blocking solution (Tris-buffered saline with 1% bovine serum albumin, 100 μM biotin, and 0.05% NaN_3_) for 40 min. The blocked array wells were aspirated and centrifuged dry, after which they were stored at 4°C.

### Microarray immunoassay procedure.

All serum samples were tested in duplicates. To measure interassay reproducibility, each test plate included negative- and positive-control samples. The negative control consisted of a pool of seronegative child sera against all tested viral antigens. The positive control consisted of a pool of highly positive child sera against all tested viral antigens. The control sera were stored in aliquots at 1:100 dilutions at −20°C, and a fresh aliquot was used in each assay. The serum samples were tested at 1:100 dilutions after analysis in different dilutions. The samples and controls were diluted in assay buffer (50 mM Tris base [pH 7.75], 150 mM NaCl, 0.05% NaN_3_, 0.01% Tween 40, 0.05% bovine gammaglobulins, 20 μM diethylenetriaminepentaacetic acid, 0.5% bovine serum albumin [BSA], 20 μg/ml of cherry red, and 1% fetal bovine serum [FBS]). Microarray plates were prewashed with a washing buffer (50 mM Tris-HCl [pH 7.75], 0.15 M NaCl, 0.05% [wt/vol] NaN_3_, 0.1% [wt/vol] gelatin, 0.01% [wt/vol] Tween 40, and 20 μM diethylenetriaminepentaacetic acid). Briefly, 50-μl volumes of 1:100 diluted serum samples were added in duplicates into array wells and incubated for 2 h at room temperature. After incubation, the wells were washed three times with washing buffer. To detect IgG antibodies bound to solid-phase antigens, anti-human IgG-coated UCNPs were diluted in assay buffer (0.03 mg/ml), thoroughly vortexed, and sonicated (Vial Tweeter block sonicator [100 A, 0.5 s, and 7 cycles]) to disperse aggregates. Then 50 μl/well of anti-hIgG-coated UCNPs was added into the wells and incubated for 40 min at room temperature with slow shaking. After four washes, the plates were dried for 2 h and the wells were imaged with an anti-Stokes photoluminescence imager constructed for multianalyte detection (19). The emission of UCNPs was detected through a 650-nm short-pass filter (Chroma Technology, Rockingham, VT) with a cooled charge-coupled-device (CCD) camera (Andor Clara with Sony ICX285 CCD; Andor Technology, South Windsor, CT) using 2× binning, a 2.2-s exposure time per well, and laser excitation at 976 ± 2 nm with 7 W of optical power.

### Image analysis program.

Image analysis was performed with ImageJ software version 1.43n. The computer program was written in Visual Basic language to aid in microarray analysis. Although existing software solutions were available (29, 30), a more flexible and alterable analysis platform was desired. The program was designed to semiautomatically address, segment, and quantitate the spots from a batch of microarray images that had the same array specifications and spot radii. The array specifications, including row and column counts, were used as input information to estimate the position, rotation, and scale of the array grid with the Radon transform (31, 32). Spots were segmented from the estimated positions using a fixed-size circular area where the radius was the average of all the detected spot boundary radii in the batch of microarray images. The final positioning and radius adjustments were done manually in the program. The computer program read unsigned 16-bit grayscale raw images where the intensity value of a single pixel ranged from 0 to 65,535. An average pixel intensity, standard deviation, and minimum and maximum intensities were automatically calculated from each circular spot area, and no automatic background subtraction was calculated or directly applied.

### Data analysis.

The antibody concentration, expressed as EIA and MAIA units, was calculated from the linear plot of the calibrators using Microsoft Excel version 2010 (Microsoft Corp., USA). Data depicted on box plots were analyzed and graphed using Origin version 2016 (OriginLab, USA). All other statistical analyses were performed using IBM SPSS Statistics version 22 software (IBM Corp., USA). Differences between groups were analyzed by independent-sample *t* test, where significance was set at a *P* value of <0.05. Agreement between EIA and MAIA was analyzed using ROC analysis, Cohen’s κ statistic, and Pearson correlation.
